# Safety, tolerability, pharmacokinetic characteristics, and immunogenicity of MW33: a Phase 1 clinical study of the SARS-CoV-2 RBD-targeting monoclonal antibody

**DOI:** 10.1080/22221751.2021.1960900

**Published:** 2021-08-18

**Authors:** Xianmin Meng, Peipei Wang, Yanqing Xiong, Yijun Wu, Xiaoyan Lin, Song Lu, Ruowan Li, Bei Zhao, Jing Liu, Shaoqing Zeng, Liyan Zeng, Yan Wu, Yan Lu, Jinchao Zhang, Datao Liu, Shuhai Wang, Hongzhou Lu

**Affiliations:** a Shanghai Public Health Clinical Center, Fudan University, Shanghai, People’s Republic of China; b Mabwell (Shanghai) Bioscience Co., Ltd., Shanghai, People’s Republic of China

**Keywords:** SARS-CoV-2, COVID-19, MW33 injection, monoclonal antibody, Phase 1 clinical trial, safety, pharmacokinetic characteristics

## Abstract

MW33 is a fully humanized IgG1κ monoclonal neutralizing antibody, and may be used for the prevention and treatment of coronavirus disease 2019 (COVID-19). We conducted a randomized, double-blind, placebo-controlled, single-dose, dose-escalation Phase 1 study to evaluate the safety, tolerability, pharmacokinetics (PK), and immunogenicity of MW33. Healthy adults aged 18–45 years were sequentially enrolled into the 4, 10, 20, 40, and 60 mg/kg dose groups and infused with MW33 over 60 ± 15 min and followed for 85 days. All 42 enrolled participants completed the MW33 infusion, and 40 participants completed the 85-day follow-up period. 34 participants received a single infusion of 4 (*n *=* *2), 10 (*n *=* *8), 20 (*n *=* *8), 40 (*n *=* *8), and 60 mg/kg (*n *=* *8) of MW33. 27 subjects in the test groups experienced 78 adverse events (AEs) post-dose, with an incidence of 79.4% (27/34). The most common AEs included abnormal laboratory test results, vascular and lymphatic disorders, and infectious diseases. The severity of AEs was mainly Grade 1 (92 AEs), and three Grade 2 and one Grade 4. The main PK parameters, maximum concentration (*C*_max_), and area under the concentration-time curve (AUC_0–*t*_, and AUC_0–∞_) in 34 subjects showed a linear kinetic relationship in the range of 10–60 mg/kg. The plasma half-life was approximately 25 days. The positive rates of serum ADAs and antibody titres were low with no evidence of an impact on safety or PK. In conclusion, MW33 was well-tolerated, demonstrated linear PK, with a lower positive rate of serum ADAs and antibody titres in healthy subjects.

**Trial registration:**ClinicalTrials.gov identifier: NCT04427501.

**Trial registration:**ClinicalTrials.gov identifier: NCT04533048.

**Trial registration:**ClinicalTrials.gov identifier: NCT04627584.

## Introduction

Coronavirus disease 2019 (COVID-19), caused by severe acute respiratory syndrome coronavirus 2 (SARS-CoV-2), is currently spreading worldwide, and poses a serious threat to mankind and the stagnant economy [1–4]. SARS-CoV-2 is characterized by a long incubation period and strong transmissibility. It causes severe disease and high mortality in susceptible individuals, particularly populace with underlying diseases [5]. While taking into account the current pandemic situation, the development of either vaccines or antibody agents to stop the spread of this infection, protect the vulnerable and then reboot the economy is urgently required.

Several agents have been evaluated for the treatment of COVID-19, but with mixed results [6–8]. Monoclonal antibodies are widely accepted as an efficacious option to treat this virus. In contrast to vaccines, antibodies have a clear and immediate effect during emergent interventions in patients, and a similar effect was demonstrated during the development of drugs for the Ebola outbreak [9]. The specificity and high affinity of monoclonal antibodies can preemptively bind to the viral S protein, disrupting its interaction with the receptor binding domain (RBD) of angiotensin converting enzyme 2 (ACE2) receptor [10–12]. According to the nowadays’ immunological theories and evidence, fully humanized neutralizing antibodies derived from the memory B cells of recovered patients have higher safety and stability than those derived by other techniques (e.g. immune hybridoma technology and natural phage antibody library technology) [11]. Since the outbreak of COVID-19, huge efforts have been made to fight against this pandemic, and develop therapeutic antibodies for SARS-CoV-2 [13,14]. LY-CoV555 (Bamlanivimab), jointly developed by Eli Lilly and AbCellera, is the first monoclonal antibody for the treatment of COVID-19, and the Phase 2 study (NCT04427501) in patients with mild or moderate COVID-19 has been completed [15,16]. The U.S. Food and Drug Administration (FDA) has issued an Emergency Use Authorization (EUA) to permit the timely use of the unapproved product LY-CoV555 for the treatment of patients with mild to moderate COVID-19. In addition, Regeneron has contacted the FDA/EUA for approval of the antibody cocktail Casirivimab + Imdevimab [17].

MW33 is a SARS-CoV-2 RBD-targeting monoclonal antibody, one of the IgG1κ subtypes, and has high neutralization activity by disrupting the interaction of the RBD with the ACE2 receptor. FcγRIIB was confirmed to be involved in the antibody-dependent enhancement (ADE) of SARS-CoV-2 infection mediated by MW33. Attributed to introducing the LALA mutation into the Fc region (MW33/LALA), it thereby completely deletes the ADE activity. Preclinical studies in rhesus monkeys showed that the MTD and NOAEL were both 400 mg/kg, dozens of times versus the counterpart of the recommended starting dose (4mg/kg) in human clinical studies, indicating a large safety margin. Potent prophylactic effects against SARS-CoV-2 were observed in rhesus monkeys. A single dose of MW33 blocked infection by SARS-CoV-2 during prophylactic treatment and cleared SARS-CoV-2 in three days in a therapeutic treatment setting. The discovery of MW33 and its effects against SARS-CoV-2 in rhesus monkeys has been reported previously [11]. These studies have been the premise of formulating preclinical evidence that MW33 may be an agent in treating COVID-19.

In this Phase 1 study, the safety, tolerability, pharmacokinetics (PK), and immunogenicity of MW33, and pilot screening for potential induction of ADAs in healthy subjects were evaluated.

## Materials and methods

### Study design and participants

In this first-in-human Phase 1, randomized, double-blind, placebo-controlled, single-dose, dose-escalation clinical trial of the humanized monoclonal antibody MW33, eligible participants were healthy adults aged 18–45 years as defined by the inclusion and exclusion criteria related to medical history, physical examination, and clinical laboratory tests, with no participation in any test drugs within the last three months. The subjects were sequentially enrolled into groups according to a protocol specified dose-escalation scheme. Human immunodeficiency virus type 1 (HIV-1), treponema pallidum particle agglutination assay (TPPA), hepatitis B and C were examined with negative results and none of the subjects weighed less than 50 kg for males and 45 kg for females. The study was registered with ClinicalTrials.gov, NCT04533048, and was conducted at the Shanghai Public Health Clinical Center. The protocol was reviewed and approved by the institutional review board (IRB) of the centre. The China Department of National Medical Products Administration (NMPA) human experimental guidelines for clinical trials were followed. Informed consent was acquired from each subject before enrolment following IRB agreement.

MW33 is a recombinant fully humanized IgG1κ monoclonal antibody which targets the S1 RBD of S protein of SARS-CoV-2 with high affinity, thereby preventing the S protein from binding to ACE2 on the host cell surface. MW33 was obtained from the blood cells of rehabilitation patients after B cell screening and single cell sequencing techniques, and manufactured by Mabwell (Shanghai) Bioscience Co., Ltd. in compliance with the Good Manufacturing Practice regulations. The preparation process of MW33 was as follows. The SARS-CoV-2 RBD, SARS-CoV-2 S1(1-685aa, accession number: QHD43416.1), and SARS-CoV-2 RBD mutants recombinant proteins tagged with C-terminal 6× His were cloned into the pKN293E expression vector. HEK293 cells were transiently transfected with plasmids using 293fectinTM Transfection Reagent (Cat:12347019, Life Technologies) when the cell density reached 1×10^6^ cells/ml. Four days after transfection, the conditioned media was collected by centrifugation followed by purification using HisTrapTM HP (Cat:17-5248-01, GE Healthcare). The purified protein was buffer exchanged into PBS using a Vivacon 500 concentrator (Cat:VS0122, Sartorius Stedim). For the generation of human ACE2-hFc and SARS-CoV-2 RBD-mFc recombinant proteins, RBD or ACE2 sequence (1-615aa, accession number: NP_068576.1) was cloned into mouse IgG1 or human IgG1 Fc backbone in pKN293E expression vectors and transiently transfected into HEK293 cells followed by media collection and purification using MabSelect SuRe antibody purification resin (Cat: 29-0491-04, GE Healthcare). SEC-HPLC and SDS-PAGE were used to check the size and purity of these recombinant proteins.

MW33 is supplied in a glass vial at 100 mg/5 mL per vial for injection. Long-term storage temperature was 2–8°C and the product was protected from light. Stability testing also showed that MW33 remains stable at 25°C for up to 6 months. Total volume of the MW33 injection was calculated according to the subject's body weight and dose. The same volume of saline from a bag containing 250 mL saline was discarded, and MW33 was added to the bag for intravenous infusion within 60 ± 15 min.

### Study procedures

Five doses of 4, 10, 20, 40, and 60 mg/kg MW33 were administered intravenously in the dose-escalation scheme. A single-arm study was conducted in the 4 mg/kg group, and 2 subjects received MW33. In addition, a randomized, controlled, double-blind study was conducted in the 10, 20, 40, and 60 mg/kg groups using a block randomization method, and the subjects in each dose group were randomized at a ratio of 4:1 to MW33 or placebo. The higher dose was not administered until the safety evaluation in all subjects in the prior dose group was complete 3 days post-dose. Each subject received only one dose. Intravenous infusions of MW33 were administered via a peripheral vein over 60 ± 15 min. The subjects were admitted to the ward one day before MW33 administration and monitored by study clinicians until discharge 168 h after administration.

Vital signs such as pulse rate, respiration rate, blood pressure, and body temperature prior to administration and after infusion were respectively assessed as per the predefined protocol. ECGs, physical examination, laboratory tests including hematology, urinalysis, blood chemistry, and coagulation function were performed prior to the product administration and after infusion on days 3, 8, 15, 29, 57, and 85. All adverse events (AEs) were recorded during the 85-day period after MW33 administration, and serious adverse events (SAEs) were assessed throughout the trial. AEs were coded using the Medical Dictionary for Regulatory Activities (MedDRA 23.0). The numbers and incidence of AEs were denoted in frequency. Total follow-up duration was 85 days after MW33 administration.

Blood samples for PK assessment of MW33 were collected at baseline (pretreatment), then on day 1 at 0.5 h (during infusion), 1 h (immediately after the end of dosing), 2, 6, 24 and 48 h post-dose, and on day 5, 8, 15, 22, 29, 43, 57, 71 and 85 or study discontinuation. MW33 serum concentrations were determined by a validated enzyme-linked immunosorbent assay (ELISA) method with a SpectraMax Plus 384 (Molecular Devices, USA). The Biotin-MW33-SIRBD-His protein was pre-coated on a NUNC Immobilizer Streptavidin F96 plate as the capture reagent, the drug in the serum sample was captured on the plate and then the plate was washed to remove the unbound material. Mouse anti-human IgG Fc antibody [HRP] was added, and peroxidase and TMB (3, 5′, 5, 5′-tetra-methylbenzidine) substrate were used to develop the colour reaction. Sulphuric acid was added to discontinue the colour reaction. Plates were read at 450 nm/630 nm with a SpectraMax Plus 384 microplate reader. MW33 concentrations were quantified using linear regression of the MW33 standard curve covering the range of 400–128,000 ng/mL.

Anti-MW33 antibodies in serum were assessed at baseline and on day 15, 29, 57 and 85 or study discontinuation. A validated Meso Scale Discovery (MSD) electrochemiluminescence (ECL) with MESO® SECTOR S600 (Analytik Jena AG, Germany) homogenous bridging assay was used for the determination of anti-MW33 antibodies in all human serum samples. Serum samples were acidified before loading into each well and incubated with biotin-labeled MW33 and sulfotag-labeled MW33 to form the drug–anti-drug antibody–drug complex for quantification. The complex was then bound to a MSD GOLDTM 96-well Streptavidin plate. The signals generated were proportional to the concentration of anti-MW33 antibody in serum samples collected pre-dose (0 h) and at 85 days from all subjects. Sample concentrations were quantified using linear regression of a MW33 standard curve covering a range of 400–12,800 ng/mL. The serum from 42 subjects was evaluated.

### Outcomes

The primary endpoints were the safety and tolerability of MW33. The secondary endpoints were PK and assessment of ADAs to MW33. Safety outcomes included the number and incidence of AEs and SAEs, clinical laboratory changes, vital signs, physical findings, and other observations related to safety.

### Statistical analysis

Pharmacokinetic analysis was performed using WinNonlin 8.2 software, and the statistical analysis was performed using SAS 9.4 software.

This is the first-in-human study, and thus the sample size in each dose group was not based on a formal power calculation. According to the “Technical Guidelines for Clinical Pharmacokinetic Research of Chemical Drugs” issued by NMPA, the enrolment of 8–12 subjects is recommended in each dose group for a single dose administration trial. Forty-two subjects were predefined and assigned to the following 5 dose-escalation groups: 4, 10, 20, 40, and 60 mg/kg. Two subjects were enrolled in the first dose group of MW33 at 4 mg/kg, and 10 subjects (8 subjects in the MW33 group and 2 subjects in the placebo group) were enrolled into the 10, 20, 40, and 60 mg/kg dose groups, respectively.

The PK parameters in each subject were calculated in accordance with the actual sampling time using a noncompartmental model, including maximum concentration (*C*_max_), area under the concentration-time curve (AUC_0–*t*_, AUC_0–∞_), time to maximum concentration (*T*_max_), elimination half-life (*t*_1/2_), clearance (CL), apparent volume of distribution (*V_z_*), and elimination rate constant (*λ_z_*). The linear relationship between the dose and the PK parameters was further evaluated using the Power model method. The linear regression relationships between the main PK exposure parameters (*C*_max_, AUC_0–*t*_ and AUC_0–∞_) and the doses were plotted, and the corresponding slopes were calculated. The positive rate of ADAs in serum was showed by descriptive analysis.

### Role of the funding source

There was no funding source for this study. All authors had full access to all the study data and had final responsibility for the decision to submit for publication.

## Results

**Figure 1.**
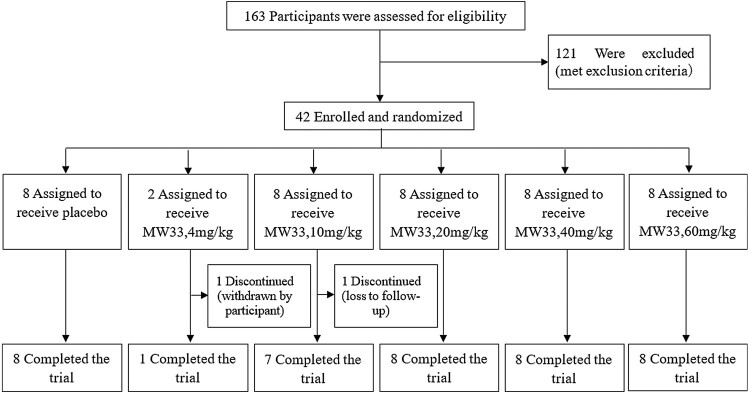
CONSORT diagram for the present trial.

From 10 August 2020 to 16 November 2020, a total of 163 participants were screened and 121 were exclusive according to the criteria. The main exclusive reasons were recorded as follow, 36 subjects of abnormal laboratory tests, 26 of abnormal vital signs, 23 of unqualified BMI, 12 abnormal chest radiography, 8 ECG abnormalities, 6 of abnormal B-mode ultrasound, 3 unqualified medication history, and 7 of the other. 42 were finally eligible and enrolled, eight were assigned to the placebo and 34 to the test groups and received one dose of MW33 (Figure 1). The demographics of the test groups (Table 1) consisted of 6 (17.7%) women and 28 (82.4%) men with a mean age of 27.6 ± 6.8 years (range 18–43 years), mean body weight of 62.2 ± 5.8 kg (range 53.6–79.4 kg), and a mean body mass index of 22.0 ± 1.5 kg/m^2^ (range 19.1–24.2 kg/m^2^). Forty of 42 subjects completed all the tests and follow-ups specified in the protocol. One subject in the 4 mg/kg group withdrew from the study voluntarily on day 71 post-dose. Another subject withdrew from the 10 mg/kg group on day 57 post-dose due to “craniocerebral injury” which was the result of an out-of-hospital accident, and was lost from follow-up (Figure 1).

**Table 1.** Demographic data and baseline characteristics of the subjects in each dose group.

	Placebo group (*n *=* *8)	Test group					
		4 mg/kg (*n *=* *2)	10 mg/kg (*n *=* *8)	20 mg/kg (*n *=* *8)	40 mg/kg (*n *=* *8)	60 mg/kg (*n *=* *8)	Total (*n *=* *34)
**Age (years)**	** **						
Mean ± SD	28.5 ± 4.7	35.0 ± 4.2	27.1 ± 6.2	30.6 ± 8.7	26.8 ± 4.5	24.1 ± 6.4	27.6 ± 6.8
Range	24–36	32–38	18–35	21–43	23–36	18–37	18–43
**Sex**							
Male	7 (87.5%)	2 (100%)	5 (62.5%)	7 (87.5%)	6 (75.0%)	8 (100%)	28 (82.4%)
Female	1 (12.5%)	0 (0%)	3 (37.5%)	1 (12.5%)	2 (25.0%)	0 (0%)	6 (17.7%)
**Ethnicity**							
Han	8 (100%)	2 (100%)	8 (100%)	8 (100%)	8 (100%)	6 (75.0%)	32 (94.1%)
Others	0 (0%)	0 (0%)	0 (0%)	0 (0%)	0 (0%)	2 (25.0%)	2 (5.9%)
**Weight (kg)**							
Mean ± SD	59.8 ± 5.8	59.2 ± 5.4	61.9 ± 7.8	61.5 ± 4.6	62.3 ± 7.9	63.7 ± 2.7	62.2 ± 5.8
Range	50.7–66.7	55.4–63.0	54.0–77.1	53.6–67.0	55.4–79.4	60.8–67.5	53.6–79.4
**BMI (kg/m^2^)**	** **						
Mean ± SD	22.0 ± 1.3	21.7 ± 1.7	22.5 ± 1.0	22.2 ± 1.8	22.0 ± 1.6	21.4 ± 1.9	22.0 ± 1.5
Range	20.8–23.9	20.5–22.9	20.7–23.6	19.6–24.2	19.1–24.1	19.6–24.0	19.1–24.2

The mean actual drug exposure of subjects in each dose group (4, 10, 20, 40, 60 mg/kg) was 233.8 ± 22.3, 612.1 ± 70.5, 1240.6 ± 93.2, 2473.6 ± 272.6, and 3634.8 ± 308.9 mg, respectively. The highest dosage was 3942 mg. The safety set was actually reporting all the 42 subjects. 81.0% (34/42) of subjects who received MW33 reported 96 AEs during treatment. In contrast, 87.5% (7/8) of subjects who received placebo reported 18 AEs; 79.4% (27/34) of those who received MW33 experienced 78 AEs post-dose (Table 2). Ninety-two AEs (95.8%) were Grade 1 in severity. Grade 2 AEs occurred in the placebo group, 20, and 40 mg/kg dose groups, respectively, and were considered unlikely to be related to MW33. One Grade 4 SAE occurred in the 60 mg/kg dose group and was judged by the investigator to be unrelated to MW33 (Table 2).

**Table 2.** AEs, and their severity and outcome in the subjects in each dose group.

				Test group											
SOC/PT		Placebo group (*n *=* *8)		4 mg/kg group (*n *=* *2)		10 mg/kg group (*n *=* *8)		20 mg/kg group (*n *=* *8)		40 mg/kg group (*n *=* *8)		60 mg/kg group (*n *=* *8)		Total (*n *=* *34)	
		No. of AEs	No. of subjects (%)	No. of AEs	No. of subjects (%)	No. of AEs	No. of subjects (%)	No. of AEs	No. of subjects (%)	No. of AEs	No. of subjects (%)	No. of AEs	No. of subjects (%)	No. of AEs	No. of subjects (%)
**Severity**	Total	18	7 (87.5%)	3	2 (100%)	15	7 (87.5%)	11	4 (50.0%)	24	7 (87.5%)	25	7 (87.5%)	78	27 (79.4%)
	Grade 1	17	7 (87.5%)	3	2 (100%)	14	7 (87.5%)	10	4 (50.0%)	23	7 (87.5%)	25	7 (87.5%)	75	27 (79.4%)
	Grade 2	1	1 (12.5%)	0	0 (0%)	0	0 (0%)	1	1 (12.5%)	1	1 (12.5%)	0	0 (0%)	2	2 (5.9%)
	Grade 3	0	0 (0%)	0	0 (0%)	0	0 (0%)	0	0 (0%)	0	0 (0%)	0	0 (0%)	0	0 (0%)
	Grade 4	0	0 (0%)	0	0 (0%)	1	1 (12.5%)	0	0 (0%)	0	0 (0%)	0	0 (0%)	1	1 (2.9%)
	Grade 5	0	0 (0%)	0	0 (0%)	0	0 (0%)	0	0 (0%)	0	0 (0%)	0	0 (0%)	0	0 (0%)
**Outcome**	Resolved	18 (100%)		3 (100%)		14 (93.3%)		11 (100%)		23 (95.8%)		25 (100%)		76 (97.4%)	
** **	Unknown	0 (0%)		0 (0%)		1 (6.67%)		0 (0%)		1 (4.17%)		0 (0%)		2 (2.56%)	

Note: 2 AEs occurred before administration and were not included in the statistical description of outcome.

The types of AEs in this study mainly included abnormal laboratory test results, vascular and lymphatic disorders, and infectious diseases. The most frequently reported AE in the MW33 groups was a positive bacterial test of urine (35.3% (12/34)), whereas increased blood unconjugated bilirubin (37.50% (3/8)) was the most frequent AE in the placebo group. Increased blood bilirubin was reported in 11.76% (4/34) of subjects in the MW33 groups and in 25.00% (2/8) in the placebo groups. Defective intraventricular conduction was reported in 2.9% of subjects (1 of 34) in the MW33 groups and in 12.5% of subjects (1 of 8) in the placebo groups. In addition, one subject (1/8) in the 60 mg/kg group experienced pruritus and rash, which was Grade 1 in severity. The AEs experienced in each group are presented in Table 3. There were no deaths, suspected unexpected serious adverse reactions (SUSAR), or study discontinuations attributed to AEs during the study.

**Table 3.** Incidence of AEs by system organ classes (SOCs).

	Test group													
SOCs/PTs	Placebo group (*n *=* *8)		4 mg/kg group (*n *=* *2)		10 mg/kg group (*n *=* *8)		20 mg/kg group (*n *=* *8)		40 mg/kg group (*n *=* *8)		60 mg/kg group (*n *=* *8)		Total (*n *=* *34)	
	No. of AEs	No. of subjects (%)	No. of AEs	No. of subjects (%)	No. of AEs	No. of subjects (%)	No. of AEs	No. of subjects (%)	No. of AEs	No. of subjects (%)	No. of AEs	No. of subjects (%)	No. of AEs	No. of subjects (%)
**Total**	18	7 (87.5%)	3	2 (100%)	15	7 (87.5%)	11	4 (50.0%)	24	7 (87.5%)	25	7 (87.5%)	78	27 (79.4%)
**Investigations**	12	7 (87.5%)	3	2 (100%)	9	6 (75.0%)	8	3 (37.5%)	21	7 (87.5%)	20	6 (75.0%)	61	24 (70.6%)
Positive bacterial test	1	1 (12.5%)	2	2 (100%)	3	3 (37.5%)	1	1 (12.5%)	4	3 (37.5%)	6	3 (37.5%)	16	12 (35.3%)
Increased blood bilirubin	3	2 (25.0%)	0	0 (0%)	0	0 (0%)	1	1 (12.5%)	1	1 (12.5%)	4	2 (25.0%)	6	4 (11.7%)
White blood cells in urine positive	0	0 (0%)	0	0 (0%)	1	1 (12.5%)	1	1 (12.5%)	4	2 (25.0%)	0	0 (0%)	6	4 (11.7%)
Increased blood unconjugated bilirubin	4	3 (37.5%)	0	0 (0%)	0	0 (0%)	1	1 (12.5%)	1	1 (12.5%)	3	2 (25.0%)	5	4 (11.7%)
Presence of ketone bodies in urine	1	1 (12.5%)	1	1 (50.0%)	0	0 (0%)	0	0 (0%)	3	3(37.5%)	1	1 (12.5%)	5	5 (14.7%)
Decreased white blood cell count	0	0 (0%)	0	0 (0%)	0	0 (0%)	1	1 (12.5%)	3	2 (25.0%)	0	0 (0%)	4	3 (8.8%)
Electrocardiogram ST segment abnormal	1	1 (12.5%)	0	0 (0%)	1	1 (12.5%)	2	1 (12.5%)	0	0 (0%)	0	0 (0%)	3	2 (5.9%)
Increased conjugated bilirubin	0	0 (0%)	0	0 (0%)	0	0 (0%)	0	0 (0%)	0	0 (0%)	3	1 (12.5%)	3	1 (2.9%)
Increased blood uric acid	1	1 (12.5%)	0	0 (0%)	1	1 (12.5%)	0	0 (0%)	1	1 (12.5%)	0	0 (0%)	2	2 (5.9%)
Increased blood triglyceride	0	0 (0%)	0	0 (0%)	1	1 (12.5%)	0	0 (0%)	1	1 (12.5%)	0	0 (0%)	2	2 (5.9%)
Decreased platelet count	0	0 (0%)	0	0 (0%)	0	0 (0%)	0	0 (0%)	0	0 (0%)	1	1 (12.5%)	1	1 (2.9%)
Increased blood urea	0	0 (0%)	0	0 (0%)	0	0 (0%)	0	0 (0%)	1	1 (12.5%)	0	0 (0%)	1	1 (2.9%)
Increased blood creatine phosphokinase	1	1 (12.5%)	0	0 (0%)	0	0 (0%)	1	1 (12.5%)	0	0 (0%)	0	0 (0%)	1	1 (2.9%)
Electrocardiogram T wave abnormal	0	0 (0%)	0	0 (0%)	0	0 (0%)	0	0 (0%)	0	0 (0%)	1	1 (12.5%)	1	1 (2.9%)
Increased eosinophil percentage	0	0 (0%)	0	0 (0%)	1	1 (12.5%)	0	0 (0%)	0	0 (0%)	0	0 (0%)	1	1 (2.9%)
Increased urinary urobilinogen	0	0 (0%)	0	0 (0%)	0	0 (0%)	0	0 (0%)	0	0 (0%)	1	1 (12.5%)	1	1 (2.9%)
Blood in urine present	0	0 (0%)	0	0 (0%)	0	0 (0%)	0	0 (0%)	1	1 (12.5%)	0	0 (0%)	1	1 (2.9%)
Positive urinary occult blood	0	0 (0%)	0	0 (0%)	1	1 (12.5%)	0	0 (0%)	0	0 (0%)	0	0 (0%)	1	1 (2.9%)
Protein in urine present	0	0 (0%)	0	0 (0%)	0	0 (0%)	0	0 (0%)	1	1 (12.5%)	0	0 (0%)	1	1 (2.9%)
**Vascular disorders**	1	1 (12.5%)	0	0 (0%)	2	1 (12.5%)	1	1 (12.5%)	0	0 (0%)	1	1 (12.5%)	4	3 (8.8%)
Hypertension	1	1 (12.5%)	0	0 (0%)	2	1 (12.5%)	1	1 (12.5%)	0	0 (0%)	1	1 (12.5%)	4	3 (8.8%)
**Infections and infestations**	1	1 (12.5%)	0	0 (0%)	0	0 (0%)	2	2 (25.0%)	1	1 (12.5%)	0	0 (0%)	3	3 (8.8%)
Upper respiratory tract infection	1	1 (12.5%)	0	0 (0%)	0	0 (0%)	1	1 (12.5%)	1	1 (12.5%)	0	0 (0%)	2	2 (5.9%)
Conjunctivitis	0	0 (0%)	0	0 (0%)	0	0 (0%)	1	1 (12.5%)	0	0 (0%)	0	0 (0%)	1	1 (2.9%)
**Cardiac disorders**	2	1 (12.5%)	0	0 (0%)	0	0 (0%)	0	0 (0%)	0	0 (0%)	1	1 (12.5%)	1	1 (2.9%)
Sinus bradycardia	0	0 (0%)	0	0 (0%)	0	0 (0%)	0	0 (0%)	0	0 (0%)	1	1 (12.5%)	1	1 (2.9%)
Defective intraventricular conduction	2	1 (12.5%)	0	0 (0%)	0	0 (0%)	0	0 (0%)	0	0 (0%)	0	0 (0%)	0	0 (0%)
**Respiratory, thoracic and mediastinal disorders**	1	1 (12.5%)	0	0 (0%)	1	1 (12.5%)	0	0 (0%)	0	0 (0%)	1	1 (12.5%)	2	2 (5.9%)
Oropharyngeal pain	0	0 (0%)	0	0 (0%)	1	1 (12.5%)	0	0 (0%)	0	0 (0%)	1	1 (12.5%)	2	2 (5.9%)
Nasal obstruction	1	1 (12.5%)	0	0 (0%)	0	0 (0%)	0	0 (0%)	0	0 (0%)	0	0 (0%)	0	0 (0%)
**Blood and lymphatic system disorders**	0	0 (0%)	0	0 (0%)	0	0 (0%)	0	0 (0%)	2	1 (12.5%)	0	0 (0%)	2	1 (2.9%)
Anemia	0	0 (0%)	0	0 (0%)	0	0 (0%)	0	0 (0%)	2	1 (12.5%)	0	0 (0%)	2	1 (2.9%)
**Gastrointestinal disorders**	1	1 (12.5%)	0	0 (0%)	1	1 (12.5%)	0	0 (0%)	0	0 (0%)	0	0 (0%)	1	1 (2.9%)
Abdominal discomfort	0	0 (0%)	0	0 (0%)	1	1 (12.5%)	0	0 (0%)	0	0 (0%)	0	0 (0%)	1	1 (2.9%)
Nausea	1	1 (12.5%)	0	0 (0%)	0	0 (0%)	0	0 (0%)	0	0 (0%)	0	0 (0%)	0	0 (0%)
**Skin and subcutaneous tissue disorders**	0	0 (0%)	0	0 (0%)	0	0 (0%)	0	0 (0%)	0	0 (0%)	2	1 (12.5%)	2	1 (2.9%)
Pruritus	0	0 (0%)	0	0 (0%)	0	0 (0%)	0	0 (0%)	0	0 (0%)	1	1 (12.5%)	1	1 (2.9%)
Rash	0	0 (0%)	0	0 (0%)	0	0 (0%)	0	0 (0%)	0	0 (0%)	1	1 (12.5%)	1	1 (2.9%)
**General disorders and administration site conditions**	0	0 (0%)	0	0 (0%)	1	1 (12.5%)	0	0 (0%)	0	0 (0%)	0	0 (0%)	1	1 (2.9%)
Pyrexia	0	0 (0%)	0	0 (0%)	1	1 (12.5%)	0	0 (0%)	0	0 (0%)	0	0 (0%)	1	1 (2.9%)
**Injury, poisoning and procedural complications**	0	0 (0%)	0	0 (0%)	1	1 (12.5%)	0	0 (0%)	0	0 (0%)	0	0 (0%)	1	1 (2.9%)
Craniocerebral injury	0	0 (0%)	0	0 (0%)	1	1 (12.5%)	0	0 (0%)	0	0 (0%)	0	0 (0%)	1	1 (2.9%)

**Figure 2.**
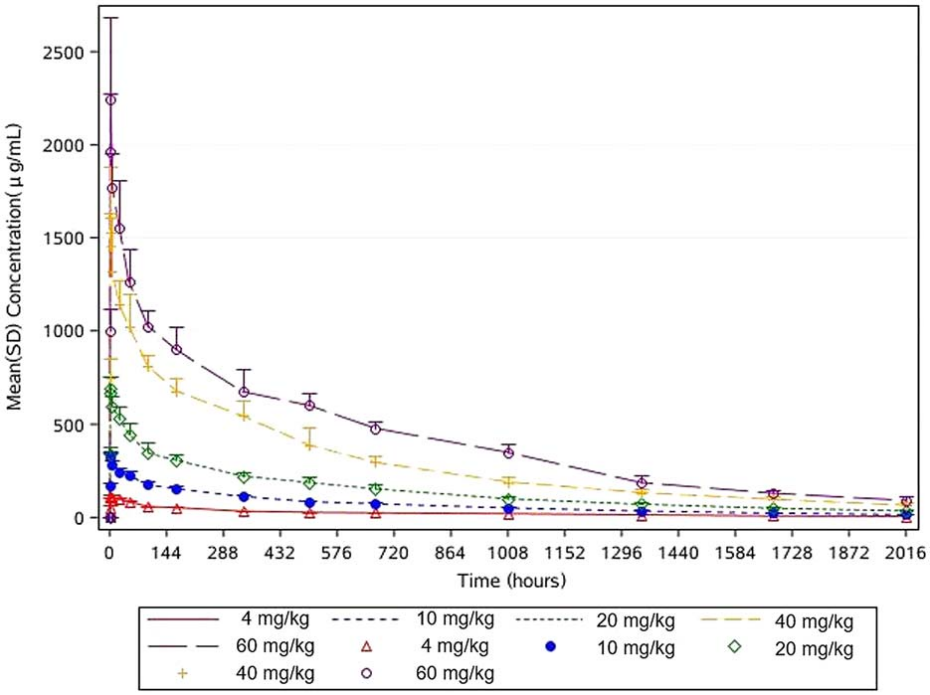
MW33 mean serum concentration-time curves of participants infused with 4 (red), 10 (blue), 20 (green), 40 (yellow), and 60 (purple) mg/kg MW33 (ordinate with standard scale, Mean + SD).

**Figure 3.**
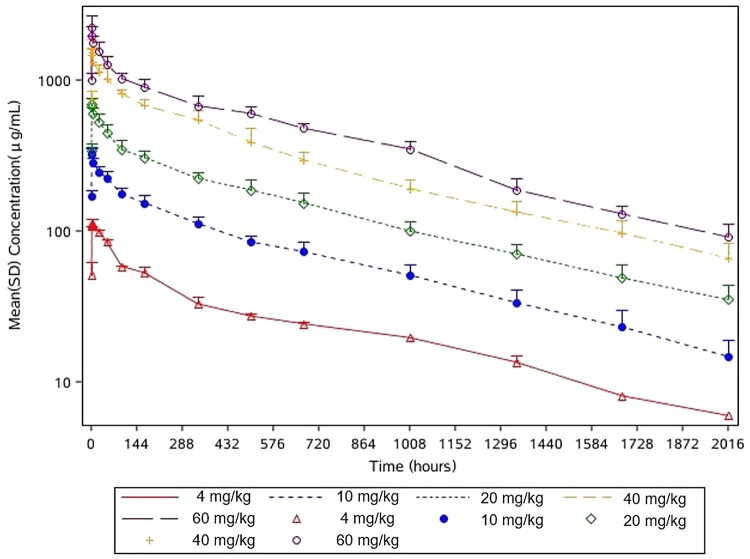
MW33 semi-logarithmic mean serum concentration-time curves of participants infused with 4 (red), 10 (blue), 20 (green), 40 (yellow), and 60 (purple) mg/kg MW33 (ordinate with Log Scale, Mean + SD).

The PK in 34 subjects were analysed, including 2 in the 4 mg/kg group, and 8 in each of the 10, 20, 40, and 60 mg/kg groups (Table 4). Mean serum concentration-time curve profiles of the participants infused with 4, 10, 20, 40, and 60 mg/kg MW33 are shown in Figure 2 and semi-logarithmic profiles are shown in Figure 3. The mean (± SD) maximum serum concentration (*C*_max_) of the intended treatment dose of 20 and 40 mg/kg were 716.55 ± 65.10 μg/mL, 1597.01 ± 314.78 μg/mL, which occurred 1.61 ± 0.52 h and 1.97 ± 1.70 h after MW33 administration, respectively. The half-life (t_1/2_) of 20 and 40 mg/kg MW33 were 26.0 ± 3.3, 24.8 ± 2.8 days respectively.

**Table 4.** Summary of the pharmacokinetic parameters of MW33 by dose group.

Statistics	4 mg/kg group (*n *=* *2)	10 mg/kg group (*n *=* *8)	20 mg/kg group (*n *=* *8)	40 mg/kg group (*n *=* *8)	60 mg/kg group (*n *=* *8)
***C*_max_ (μg/mL)**					
Mean ± SD	113.3 ± 7.2	336.3 ± 26.5	716.6 ± 65.1	1597.0 ± 314.8	2338.1 ± 402.1
CV%	6.38	7.88	9.09	19.71	17.20
Range	108.2–118.5	299.4–371.1	623.8–810.0	1122.0–2225.0	1961.0–2898.9
AUC_0–*t*_ (h*μg/mL)					
Mean ± SD	49,023.9	134,560.7 ± 16,263.9	280,948.8 ± 35,075.1	583,651.0 ± 62,067.9	831,047.1 ± 51,956.5
CV%		12.09	12.48	10.63	6.25
Range	49,023.9–49,023.9	111,910.6–154,088.3	237,188.4–334,225.8	499,530.5–698,153.7	749,453.9–902,886.9
AUC_0–∞_ (h*μg/mL)					
Mean ± SD	54,829.9	147,635.0 ± 21,311.1	313,295.4 ± 46,334.3	636,815.3 ± 72,588.2	898,312.6 ± 65,473.9
CV%		14.43	14.79	11.40	7.29
Range	54,829.9–54,829.9	118,130.9–175,027.5	254,574.8–390,663.9	531,850.0–767,327.6	795,364.0–984,779.2
***t*_1/2_ (days)**					
Mean ± SD	28.1	24.8 ± 3.6	26.0 ± 3.3	24.8 ± 2.8	22.2 ± 1.3
CV%		14.68	12.78	11.23	5.72
Range		20.4–30.6	21.1–3.3	20.5–28.0	20.7–23.8
***λ_Z_* (1/*h*)**					
Mean ± SD	0.0010	0.0012 ± 0.0002	0.0011 ± 0.0002	0.0012 ± 0.0001	0.0013 ± 0.0001
CV%		14.37	13.45	12.08	5.75
Range	0.0010–0.0010	0.0009–0.0014	0.0009–0.0014	0.0010–0.0014	0.0012–0.0014
***T*_max_ (h)**					
Mean ± SD	1.52 ± 0.68	1.22 ± 0.47	1.61 ± 0.52	1.97 ± 1.70	1.74 ± 0.49
CV%	45.09	38.59	32.27	86.68	28.24
Range	1.033–2	0.933–1.983	0.95–2	0.9–5.983	0.933–2
***V_z_* (mL/kg)**					
Mean ± SD	72.04 ± 1.61	56.91 ± 5.29	57.81 ± 5.77	53.82 ± 5.62	50.59 ± 3.47
CV%	2.24	9.29	9.98	10.44	6.86
Range	70.90–73.18	47.48–62.09	49.28–68.43	44.80–61.95	44.34–55.37
**CL (mL/h/kg)**					
Mean ± SD	0.07 ± 0.00	0.07 ± 0.01	0.07 ± 0.01	0.06 ± 0.01	0.07 ± 0.01
CV%	3.16	14.84	14.56	13.77	10.38
Range	0.07–0.08	0.06–0.08	0.05–0.08	0.05–0.08	0.05–0.08

Notes: Serum concentrations at 1008, 1680 and 2016 h post-dose were missing for S0102; serum concentrations at 1344, 1680 and 2016 h post-dose were missing for S0209; serum concentrations at 672 and 1680 h post-dose were missing for S0507; serum concentrations at 1344 h post-dose were missing for S0409. Considering that all the above blood sampling points were missing at the end of the elimination phase and had little effect on PK parameters, the PK parameters were still calculated, and AUC, *t*_1/2_ and *λ_z_* parameters were not statistically described.

With respect to the 2 subjects enrolled in the sentinel dose group of 4 mg/kg, one subject’s serum concentrations on day 42, day 70, and day 84 post-dose were missed, which may have had a large impact on the evaluation of AUC. Therefore, the linear correlation between dose and drug plasma exposure in the range of 10–60 mg/kg was analysed. The 90% confidence intervals (CIs) for slope β of the linearity equation for *C*_max_, AUC_0–*t*_, and AUC_0–∞_ were 0.9231–1.0406, 0.9475–1.0586, and 0.9398–1.0609, within the determination intervals of 0.8207–1.1798, 0.8879–1.1121 and 0.8879–1.1121, respectively. Within the dose range of 10–60 mg/kg, the PK exposure parameters *C*_max_, AUC_0–*t*_ and AUC_0–∞_ increased with escalated doses, the apparent linear PK characteristics were demonstrated (Table 5).

**Table 5.** Analysis of linear relationship of *C*_max_, AUC_0–*t*_, AUC_0–∞_ with dose (dose range: 10–60 mg/kg)-calculated by actual dose.

Parameter	*R*^2^	*α*	*β*	*P*-value	90% CI of *β* (%)	Critical region
Ln(*C*_max_)	0.9640	−2.5435E-15	0.9819	<0.0001	0.9231–1.0406	0.8207–1.1798
Ln(AUC_0–*t*_)	0.9723	0.0277	1.0031	<0.0001	0.9475–1.0586	0.8879–1.1121
Ln(AUC_0–∞_)	0.9670	0.0276	1.0003	<0.0001	0.9398–1.0609	0.8879–1.1121

Three subjects were reported to have positive ADAs. One subject in the placebo group was positive on day 1 pre-dose (baseline) and on day 15, day 57, and day 85 post-dose. One subject who received 4 mg/kg MW33 was positive only on day 15 post-dose, and one subject who received 20 mg/kg MW33 tested positive on day 29 post-dose, both of these subjects were negative at the end of the study. The antibody titre in the 4 mg/kg group was 2.0 on day 15 post-dose, and the titres in the other groups were less than 1.0. In addition, only transient ADAs were detected, and had no marked effect on PK characteristics or on AEs.

## Discussion

The COVID-19 pandemic has lasted more than one year and the number of new cases and deaths continue to rise. Similar to influenza viruses, SARS-CoV-2 may coexist with humans for a long time. Monoclonal antibodies are widely accepted as an efficacious means of preventing and treating COVID-19. Lilly's novel coronavirus neutralizing antibody LY-CoV555 (Bamlanivimab) and Regeneron's antibody cocktail therapy (Casirivimab + Imdevimab) were approved by the FDA for EUA in November 2020. SARS-CoV-2 neutralizing antibody therapy has been reported in a series of studies with evidence of a breakthrough effect in COVID-19.

The current randomized, double-blind, placebo-controlled, single-dose escalation Phase 1 study investigated the safety, tolerability, immunogenicity, and PK of MW33 in healthy subjects, and demonstrated favourable safety and PK characteristics. Forty-two subjects were enrolled in this study, all of whom completed the dosing schedule. Forty subjects actually completed all the assigned doses, and drug tolerability was acceptable and a MTD was not observed.

Most of the AEs were mild, and also the abnormal laboratory results, without symptomatic manifestations or the need for medical intervention and resolved during the follow-up period. In addition, two allergies, pruritus and rash, in the 60 mg/kg MW33 were reported. Similar AEs have also been reported following the administration of other neutralizing antibody products, and rash was reported in 3 subjects in a Phase 2 clinical study of Lilly's novel coronavirus neutralizing antibody LY-CoV555 [15,18]. The results of a pre-clinical toxicity study of MW33 showed a transient decrease in erythroid-related parameters (red blood cell, hemoglobin, hematocrit) after a single infusion. In this Phase 1 trial, only 1 subject in the 60 mg/kg group had a Grade 1 decrease in platelet count, which was possibly related to MW33. The subject recovered in the final documentation. There were no AEs leading to withdrawal from the study, no SUSARs, and no deaths. Only 1 SAE occurred and was not related to MW33. Immunogenicity was observed in two subjects, but did not result in a safety risk and did not seem to affect the PK. The safety profile in subjects who received MW33 was similar to that in placebo-treated subjects. These data imply that MW33 is probably safe.

The mean *t*_1/2_ of MW33 in the elimination phase was 28.1 days, 24.8 ± 3.6 days (CV 14.7%), 26.0 ± 3.3 days (CV 15.8%), 24.8 ± 2.8 days, (CV 15.8%), and 22.2 ± 1.3 days (CV 15.8%), respectively; CL was 0.07 ± 0.00, 0.07 ± 0.01, 0.07 ± 0.01, 0.06 ± 0.01, and 0.07 ± 0.01 mL/h/kg, respectively; the elimination rate was basically consistent across dose groups. As reported in the EUA Instructions for LY-CoV555 (Bamlanivimab) developed by Lilly [2], following a single intravenous injection of 700 mg LY-CoV555 in COVID-19 patients, the mean *t*_1/2_ was 17.6 days (with a CV of 15.8%) and the CL was 0.27 L/h (with a CV of 22.3%). The results published on its website regarding the Phase 1/2 clinical studies of REGN-COV2 (REGN10933 and REGN10987) developed by Regeneron, showed that the half-lives were 24 and 25 days after single intravenous doses of 1.2 and 4 g of REGN10933 and were 21 and 18 days after single intravenous doses of 1.2 and 4 g of REGN10933 in patients with COVID-19 [19]. In addition, mAb114 a monoclonal antibody against Ebola virus, is an IgG antibody similar to MW33. The half-lives of mAb114 after a single intravenous dose of 5, 25, and 50 mg/kg in healthy subjects were 20.1 ± 6.9, 26.7 ± 3.8, and 23.6 days, respectively [9]. Based on the above information, the half-life of MW33 was similar to other monoclonal antibodies, and LY-CoV555, REGN-COV2, and mAb114 were all administered as single doses in patients. Considering the clinical characteristics of COVID-19, it is speculated that a single intravenous injection of MW33 may be efficacious in the treatment of COVID-19.

MW33 targets the S1 RBD of SARS-CoV-2, and its metabolism and elimination in vivo are not mediated by cytochrome P450 enzymes, instead it is eliminated via proteolytic nonspecific elimination pathways. The relevant receptors exist in COVID-19 patients and could also be degraded by receptor-mediated antigen–antibody binding. Therefore, the PK profile of COVID-19 patients may be different to those of healthy subjects. Subsequent clinical studies will be conducted to further evaluate the PK profile of MW33 in the patients.

This first-in-human study mainly evaluated the potential treatment of COVID-19 by the SARS-CoV-2 neutralizing antibody MW33. The study was designed based on preclinical pharmacodynamic studies. The 20 mg/kg dose was established to be an effective prophylactic dose and 40 mg/kg was established to be an effective therapeutic dose in a rhesus monkey infection model. The main limitation of the present study is that the efficacy of MW33 was not assessed. The ongoing Phase 2 study and future studies will further investigate the additional data from COVID-19 patients.

In summary, MW33 was well-tolerated, demonstrated linear PK, with a lower positive rate of serum ADAs and antibody titres in healthy subjects after a single intravenous dose ranging from 4 to 60 mg/kg, and showed great potential for the treatment of SARS-CoV-2 infection. The main PK parameters *C*_max_, AUC_0–*t*_, and AUC_0–∞_ showed a linear kinetic relationship in the dose range of 10–60 mg/kg. The mean t_1/2_ in each dose group ranged from 533 h to 673.65 h with a similar elimination rate. The positive rates of serum ADAs and antibody titres were low in healthy subjects after a single intravenous dose of MW33, and no impact on safety or PK was observed. Based on the Phase 1 clinical trial data, a multinational randomized-controlled study (NCT04627584) is currently underway to assess the efficacy and safety of MW33 in patients with mild or moderate COVID 19. These data will pave a path to elucidate the efficacy and safety of MW33 in COVID-19 patients, and till the advent of agent, for probably uprising patients out of the impending hazard.
